# Fluorescent membrane markers elucidate the association of *Borrelia burgdorferi* with tick cell lines

**DOI:** 10.1590/1414-431X20165211

**Published:** 2016-06-20

**Authors:** R.C. Teixeira, B.A. Baêta, J.S. Ferreira, R.C. Medeiros, C.M. Maya-Monteiro, F.A. Lara, L. Bell-Sakyi, A.H. Fonseca

**Affiliations:** 1 Laboratório de Doenças Parasitárias, Instituto de Veterinária, Universidade Federal Rural do Rio de Janeiro, Seropédica, RJ, Brasil; 2 Instituto Oswaldo Cruz, Fundação Oswaldo Cruz, Rio de Janeiro, RJ, Brasil; 3 The Tick Cell Biobank, The Pirbright Institute, Pirbright, UK

**Keywords:** Borrelia burgdorferi, Tick cell lines, Phagocytosis, Fluorescent membrane marker

## Abstract

This study aimed to describe the association of *Borrelia burgdorferi* s.s. with ixodid tick cell lines by flow cytometry and fluorescence and confocal microscopy. Spirochetes were stained with a fluorescent membrane marker (PKH67 or PKH26), inoculated into 8 different tick cell lines and incubated at 30°C for 24 h. PKH efficiently stained *B. burgdorferi* without affecting bacterial viability or motility. Among the tick cell lines tested, the *Rhipicephalus appendiculatus* cell line RA243 achieved the highest percentage of association/internalization, with both high (90%) and low (10%) concentrations of BSK-H medium in tick cell culture medium. Treatment with cytochalasin D dramatically reduced the average percentage of cells with internalized spirochetes, which passed through a dramatic morphological change during their internalization by the host cell as observed in time-lapse photography. Almost all of the fluorescent bacteria were seen to be inside the tick cells. PKH labeling of borreliae proved to be a reliable and valuable tool to analyze the association of spirochetes with host cells by flow cytometry, confocal and fluorescence microscopy.

## Introduction

Lyme borreliosis (LB) is the most prevalent vector-borne bacterial disease of humans in the western world (1). LB is caused by spirochetes of the *Borrelia burgdorferi sensu lato* complex, which comprises at least *B. burgdorferi sensu stricto*, *B. afzelii*, *B. garinii*, *B. valaisiana*, *B. spielmanii*, *B. lusitaniae*, *B. bavariensis*, *B. kurtenbachii* and *B. bissettii* (2,3). Despite distinct clinical manifestations, all of these agents are transmitted by ticks of the genus *Ixodes* (3,4). Since its original description, LB has risen from relative obscurity to become a prototypal emerging infectious disease (1).

Mammalian cell cultures have provided insights into the pathogenesis of LB in the vertebrate host. Furthermore, they have supported the identification of cellular receptors for spirochete adherence in addition to various strategies for inducing an adaptive immune response against spirochetes *in vitro* (5). Similar studies using tick cells have elucidated the phenomenon of spirochete tropism within tick tissues and cells, as well as spirochete transmission mechanisms (6–8).

*Borrelia* spp. do not appear to be highly vector species-specific, although differences have been observed in their affinities for embryonic cells derived from different vector and non-vector tick species (9). The ability of these spirochetes to interact with a variety of cell types may be an important factor in their infectivity for different hosts (9). Several studies have described the interaction and phagocytosis of *Borrelia* spirochetes by tick cells; however, none of them present reliable descriptions of the early events of this phenomenon (6,8,9).

Tick cell lines have already proven to be a useful tool for studying the interactions of several economically important tick-borne pathogens with tick cells, helping to define the complex nature of the host-vector-pathogen relationship (10). The present study aimed to measure the degree of association with, and internalization of, *B. burgdorferi* strain G39/40 in eight different tick cell lines, utilizing PKH staining of *B. burgdorferi* as a powerful and reliable tool to study interaction of this pathogen with cells by flow cytometry and confocal and fluorescence microscopy.

## Material and Methods

### *Borrelia burgdorferi* strain and growth conditions

The *B*. *burgdorferi* s.s. strain G39/40 (11) was originally isolated from *Ixodes scapularis* in the USA and was kindly provided by Dr. Natalino Yoshinari of the Universidade de São Paulo, Brazil. The strain was propagated in Barbour-Stoenner-Kelly (BSK-H) medium (Sigma-Aldrich Brasil Ltda., Brazil) at 34°C and had been passaged weekly in our laboratory for more than 3 years.

To confirm the species identity, DNA was extracted from cultured spirochetes with a Qiagen DNeasy extraction kit (Qiagen, Germany), following the manufacturer's recommendations, and quantified by spectrophotometry with a NanoDrop 2000 spectrophotometer (Thermo Scientific/Sinapse Biotecnologia Ltda., Brazil). Subsequently, polymerase chain reaction (PCR) was performed according to Mantovani and collaborators (12). The reactions were performed using the following primers: *flgE* 470 Fw: 5′-CGCCTATTCTAACTTGACCCGAAT-3′ and *flgE* 470 Rev: 5′-TTAGTGTTCTTGAGCTTAGAGTTG-3′.

PCR product purification was performed with a Wizard SV Gel and a PCR Clean-up System kit (Promega, Brazil) following the manufacturer's recommendations. After purification, the amplified product was sequenced in a capillary-type platform ABI 3730 DNA Analyzer (Applied Biosystems, Life Technologies do Brasil Ltda, Brazil), and the sequences were analyzed with the Analysis 5.3.1 (CD Genomics NY, USA) program. The results were evaluated with Chromas Lite 2.01 (Thecnelysium, Pty, Ltd, Australia) program, and sequence similarities were determined by BLAST analysis of *Borrelia* spp. sequences published in GenBank.

### Tick cell lines and culture conditions

A total of 8 tick cell lines derived from the ixodid genera *Amblyomma* (AVL/CTVM17), *Hyalomma* (HAE/CTVM8), *Ixodes* (IRE/CTVM19, IDE8, ISE6) and *Rhipicephalus* (RA243, RAE/CTVM1, BME/CTVM2), were used at passage levels between 96 and 350 depending on the cell line. The tick species and instars from which cell lines were derived, and their culture media and incubation temperatures are shown in Table 1 (13–18).

**Table:**
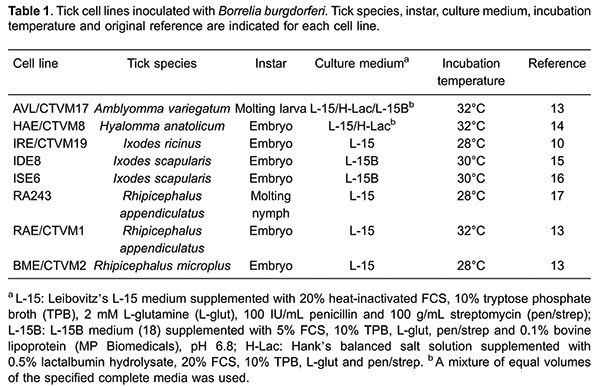

The tick cell lines were routinely maintained in sealed flat-sided tubes (Nunc, Denmark) at temperatures between 28°C and 32°C. Medium changes were performed weekly by removing and replacing approximately two-thirds of the medium volume. Subcultures were carried out by adding an equal volume of fresh complete culture medium, resuspending the cells by pipetting, and transferring half of the resultant cell suspension into a new tube.

### Staining *B. burgdorferi* with PKH67and PKH26 and flow cytometry

Spirochetes were stained with a fluorescent membrane marker, either PKH67 (green) or PKH26 (red) (Sigma-Aldrich Brasil Ltda.) as follows. A 1-mL aliquot of axenically grown *B. burgdorferi* suspension at a concentration of 4×10^7^ spirochetes/mL was washed once in Hank's balanced salt solution (HBSS). Two hundred microliters of diluent provided with the kit (Sigma-Aldrich Brasil Ltda.) and 1 μL of PKH67 or PKH26 were added to the bacterial suspension. After 10 min incubation at room temperature with periodic homogenization, 1 mL of fetal calf serum (FCS; Gibco/Life Technologies, Brazil) was added to the bacterial suspension for 1 min to stop the reaction. The suspension was centrifuged at 14,000 *g* for 5 min and resuspended in 100 μL of BSK-H medium.

Different tick cell lines were resuspended in culture medium without antibiotics and seeded at a mean of 2.7×10^5^ cells/well in 24-well plates with 6 wells for each cell line. For each cell line, cells in three of the wells were cultured in 300 µL of a 1:9 mixture of BSK-H medium and appropriate tick cell medium, and cells in the remaining 3 wells were cultured in 300 µL of a 9:1 mixture of BSK-H medium and appropriate tick cell medium. For flow cytometry, stained *B. burgdorferi* were added to the tick cells at a multiplicity of 10 bacteria to each cell in a volume of 300 μL. The plates were incubated at 30°C for 24 h without light. Tick cells incubated without spirochetes served as negative controls.

After 24 h of incubation, interactions between the bacteria and cells were stopped by washing with HBSS to remove any free spirochetes, and the cell samples were fixed by the addition of 1% paraformaldehyde and held at 4°C until analysis. After fixation, the cells were pipetted to resuspend them and the flow cytometric analyses were performed using a BD Accuri C6 cytometer (BD Biosciences, USA) with the FL1-A channel and 10,000 cells. The index of bacterial association was expressed as the percentage of fluorescent cells. Analysis of variance (ANOVA) and Tukey's test were conducted with a significance level of 5% to compare the mean percentage of fluorescent cells between each tick cell line.

### Internalization of *B. burgdorferi* by RA243 tick cell line

RA243 cells were prepared at the same concentration as before, to verify the internalization of bacteria by flow cytometry. Leibovitz's L-15 medium supplemented with 0.2% DMSO was used, either alone or supplemented with 0.2% cytochalasin D (1 mg/mL in DMSO) to inhibit phagocytosis. After 1 h, *B. burgdorferi* stained with PKH67 were added at a multiplicity of 10 bacteria to each cell.

After 24 h of incubation at 30°C, the cultures were washed with HBSS to remove free spirochetes, and the samples were resuspended in HBSS with 10% heat-inactivated FCS. The samples were analyzed by flow cytometry using channel FL1-A, and 10,000 cells were collected. The index of bacterial association was expressed as the percentage of fluorescent cells.

After the first flow cytometry analysis, the samples were stained with trypan blue (1%) and reanalyzed. The quenching effect of trypan blue on extracellular fluorescence was used to differentiate spirochete attachment from uptake.

### Fluorescence microscopy

All tick cell lines were cultured at a mean of 2.7×10^5^ cells/well on coverslips in 24-well plates. The same protocol that was used to stain the spirochetes with PKH67 or PHK26 and inoculate them into tick cell cultures for flow cytometry was used for fluorescence microscopy.

After 24 h incubation at 30°C without light, each well was gently washed with HBSS and fixed with 500 μL of 4% paraformaldehyde for 20 min. The wells were washed with HBSS and the nuclei were stained with 4′,6-diamidino-2-phenylindole (DAPI, Sigma-Aldrich Brasil Ltda.). The stained coverslips were held in the wells with HBSS at 4°C without light. At the time of analysis, the coverslips were removed from the wells and placed on slides for viewing on a Zeiss Axio Observer.Z1 fluorescence microscope (Carl Zeiss, Germany), operated by Axiovision software (Carl Zeiss). Images were acquired with a CCD camera using the filter set Zeiss 50 and 60, excited by Colibri illumination system with LED 530 and 390 nm, respectively. Optical slices were acquired using an Olympus FV1000 Confocal microscope (Olympus, Japan). Images were processed and edited using Photoshop v.4.0 (Adobe Systems, USA).

## Results

### Confirmation of *Borrelia* species identity

The spirochete isolate was confirmed as *B. burgdorferi*. The sequence amplified from the *flgE* flagellin gene of *B. burgdorferi* s.s. aligned with several species of the genus *Borrelia*; however, it had the greatest degree of sequence identity with *B. burgdorferi*, and it shared 100% identity with the sequences BORFLAA (Genbank M67456.1), BOR1FLA (Genbank L42876.1) and BORFLAA (Genbank M67456.1), among others.

### Flow cytometry

PKH stained *B. burgdorferi* efficiently without affecting bacterial viability or motility when observed in dark field after 24 h, retaining approximately 95% motility. The use of PKH staining allowed the subsequent quantification of spirochete association with 8 tick cell lines by flow cytometry and by fluorescence and confocal microscopy.

**Figure 1.**
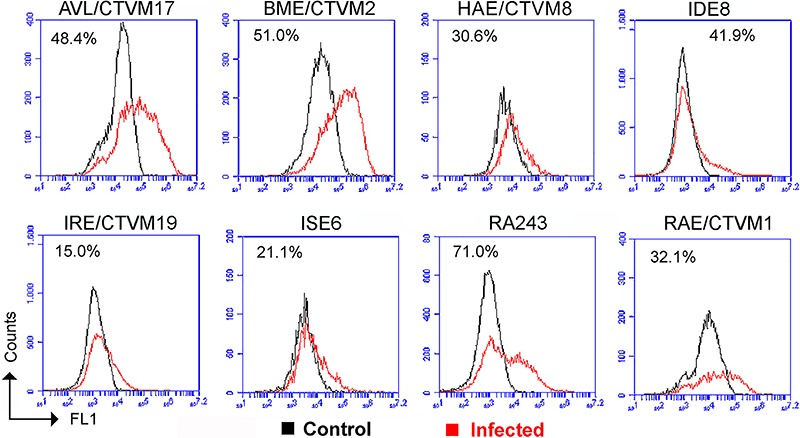
Flow cytometry overlays showing the association between *Borrelia burgdorferi* stained with PKH67 and the tick cell lines AVL/CTVM17, BME/CTVM2, HAE/CTVM8, IDE8, IRE/CTVM19, ISE6, RA243 and RAE/CTVM1 in medium containing 10% BSK-H + 90% tick cell medium. Red lines represent the cultures inoculated with *B. burgdorferi*; black lines represent the uninfected cell controls. The values inside the graphs are the mean percentage of cells with internalized spirochetes.

After 24 h of co-cultivation, *B. burgdorferi* fluorescence could be detected by flow cytometry (Figure 1), indicating a high internalization of this strain by all 8 tick cell lines. According to the flow cytometry analyses, the *R. appendiculatus* cell line RA243 achieved the best results, with 71% fluorescent cells, followed by BME/CTVM2 with 51% and AVL/CTVM17 with 48% (Figure 1). The tick cell line IRE/CTVM19 from *I. ricinus* (known to be a natural vector of *B. burgdorferi*) showed the lowest average percentage (15%) of fluorescent cells (Figure 1).

**Figure 2.**
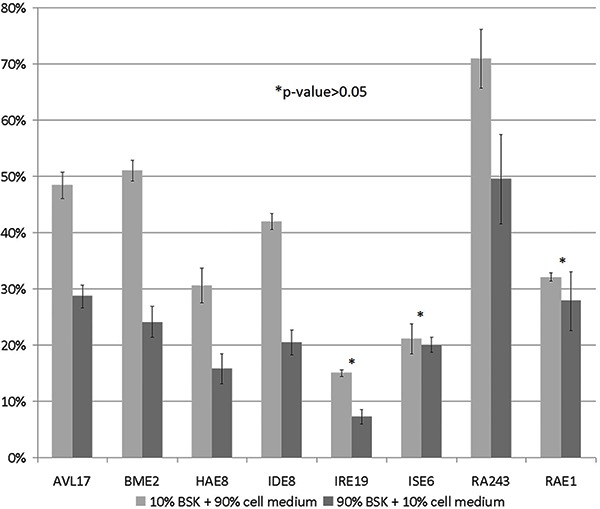
Percentage of tick cells with attached or internalized spirochetes as determined by flow cytometry. The tick cell lines AVL/CTVM17 (AVL17), BME/CTVM2 (BME2), HAE/CTVM8 (HAE8), IDE8, IRE/CTVM19 (IRE19), ISE6, RA243 and RAE/CTVM1 (RAE1) were inoculated with *Borrelia burgdorferi*, stained with PKH67, and cultivated in two different concentrations of medium, namely 10% BSK-H medium + 90% tick cell medium and 90% BSK-H medium + 10% tick cell medium. Data are reported as mean ± SD. There were no significant differences (*P>0.05) for the IRE19, ISE6 and RAE1 cell lines between the two combinations of culture media (ANOVA).

The optimum media for maintaining *B. burgdorferi* and tick cells are very different. In order to determine any effect of this change of environment on the infectivity of the bacteria, the analysis was performed in both media. The results are shown in Figure 2. Different concentrations of BSK-H medium significantly influenced the proportions of spirochete-cell associations detected by flow cytometry, without changing the overall pattern in the different cell lines. Cultures containing lower concentrations of BSK-H favored the attachment and/or entry of spirochetes into cells. All tick cell lines showed a higher percentage of fluorescent cells in media containing 10% BSK-H (Figure 2), indicating a cell-dependent process. However, the statistical analysis showed no significant difference (P>0.05) in the IRE/CTVM19, ISE6 and RAE/CTVM1 cell lines between the two combinations of culture media.

The fluorescent membrane markers PKH67 and PKH26 did not cause deleterious effects on the viability or motility of bacteria; in addition, they exhibited strong fluorescence and the ability to maintain this fluorescence for several days.

### Internalization of *B. burgdorferi* in tick cells

Treatment of the RA243 cell line (the cell line with the highest mean percentage of fluorescent cells) with cytochalasin D dramatically and significantly (P<0.05) reduced the average percentage of cells with internalized borreliae from 62% in the DMSO control to 16% in cells treated with cytochalasin D (data not shown).

The mean percentage of cells with internalized spirochetes detected by flow cytometry after trypan blue staining was not significantly different from that in the absence of trypan blue staining. This result suggests that almost all bacteria had an intracellular localization after 24 h. This finding was also observed by confocal and immunofluorescence microscopy.

**Figure 3.**
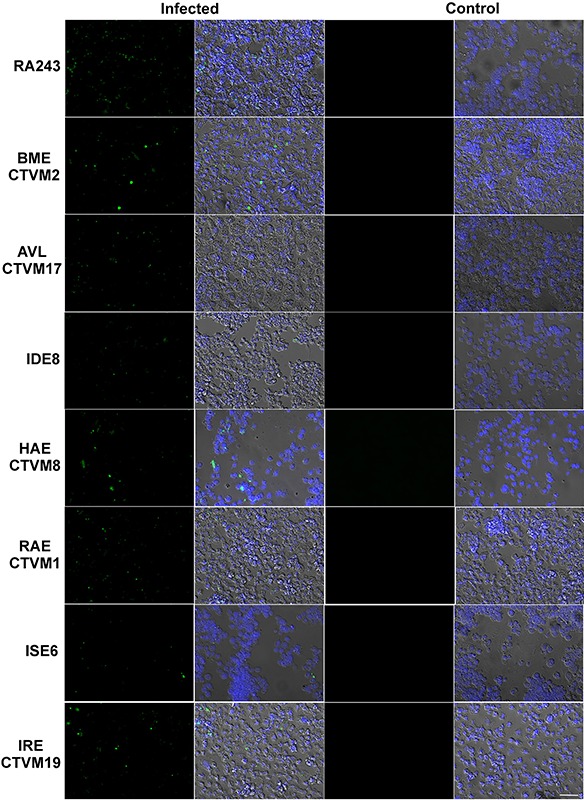
Tick cell lines RA243, BME/CTVM2, AVL/CTVM17, IDE8, HAE/CTVM8, RAE/CTVM1 (RAE1), ISE6 and IRE/CTVM19 inoculated with *Borrelia burgdorferi* previously stained with PKH67 and uninfected control cells after 24 h in 10% BSK-H + 90% cell medium. In the second and fourth columns, nuclei were stained with DAPI. The scale bar represents 50 μm.

**Figure 4.**
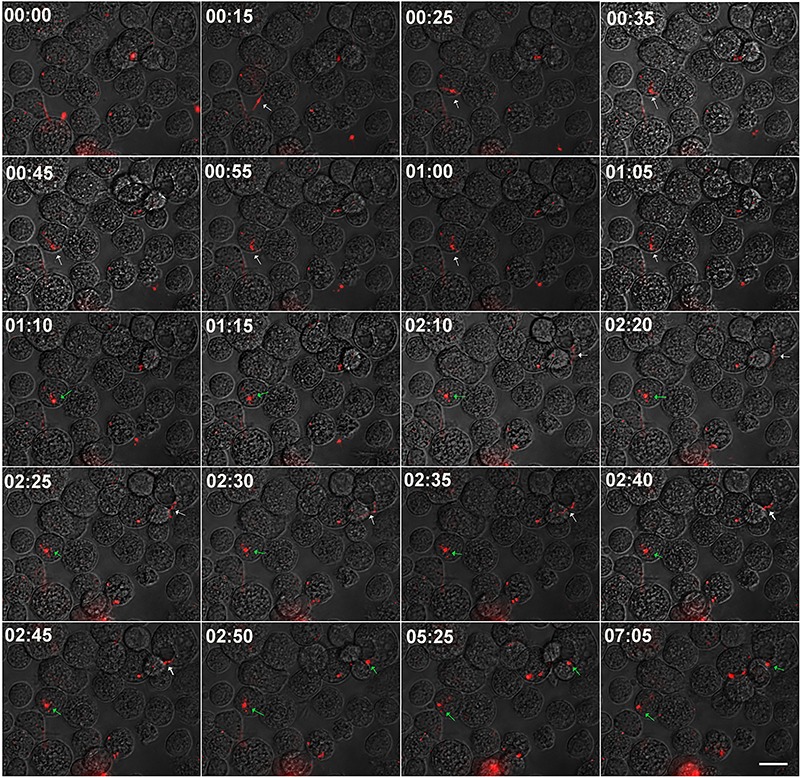
Time-lapse analysis of *Borrelia burgdorferi* uptake by AVL/CTVM17 cells. PKH26 stained *B. burgdorferi* were added at a multiplicity of 50 bacteria to each tick cell to a culture of AVL/CTVM17 cells and monitored by time-lapse microscopy during the first 7 h, with a 5 min interval. The selected images show an extracellular *B. burgdorferi* (white arrow) taking about 50 min to attach to and be internalized by a tick cell (green arrow). The scale bar represents 25 μm.

**Figure 5.**
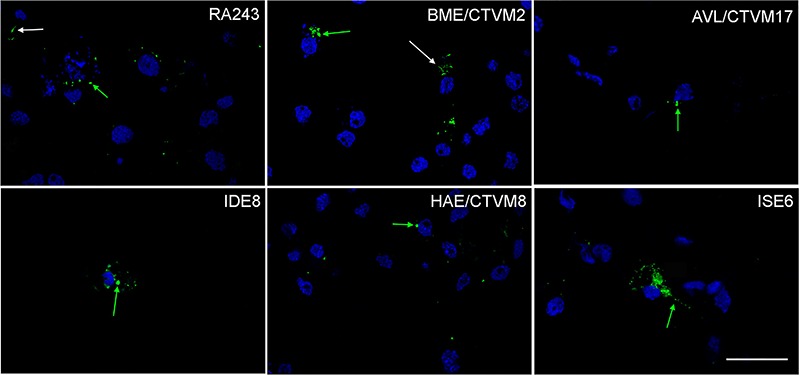
Confocal optical slices of tick cell lines 24 h after inoculation with PKH67-stained *Borrelia burgdorferi.* Internalization was performed in 10% BSK-H + 90% cell medium in cell lines RA243, BME/CTVM2, AVL/CTVM17, IDE8, HAE/CTVM8 and ISE6, as indicated. Extracellular and internalized *B. burgdorferi* are indicated by white and green arrows, respectively. Representative stacked images were taken at a defined cross-section of the tick cells as close as possible to the center of the cell. The scale bar represents 50 μm.

Microscopical analysis demonstrated that *B. burgdorferi* was rarely seen as a spiral form, whether attached to tick cells or as a free-living organism. After 24 h, almost all fluorescent bacteria were observed as intracellular dots (Figure 3). Time-lapse analysis of a representative AVL/CTVM17 culture infected with PKH26-stained spirochetes demonstrated that this phenomenon was due to a very quick attachment-invasion process, which took about 50 min to complete (Figure 4). It was, therefore, considered feasible to distinguish free or attached spirochetes, presenting elongated or helical morphology (indicated by white arrows) from intracellular spirochetes that presented rounded morphology (green arrows). To confirm these observations, after 24 h of internalization, different tick cell cultures were fixed and observed with a confocal microscope. Almost all of the fluorescent material was located inside the tick cells as seen in different transverse sections by confocal microscopy (Figure 5). This figure reveals that a few bacteria presented elongated morphology, being attached to the cell surface (white arrows), but the majority of the fluorescence appeared as a punctuate signal inside the cells (green arrows).

## Discussion

The use of fluorophores allows better visualization of bacteria in microscopy studies, and is also a technique for studying the quantitative association of bacteria with different cells. Other studies with different dyes, such as carboxyfluorescein diacetate (CFSE) and isothiocyanate isomer I (FITC), have previously been used to stain different genospecies of *B. burgdorferi* to verify the association between spirochetes and cells (8,19). Unfortunately, the emission of these fluorophores suffers a massive quenching in acidic environments (19). Genetic modification of *B. burgdorferi* to express fluorescent proteins (20) is another approach to labeling spirochetes for visualization of their interaction with cells (21). However, this technique is considerably more complex than labeling with fluorophores and the latter method has the advantage of being quickly and easily applied to any newly isolated strain of *Borrelia*.

One advantage of using labeled bacteria is the simplicity of the quantification assay. Flow cytometry is rapid and sensitive, and requires small sample volumes. Compared to microscopy, a much larger number of cells can be measured by flow cytometry, and the technique is not as prone to errors in interpretation by the operator as microscopy. However, microscopic techniques have the advantage of enabling the direct visual assessment of interactions between tick cells and spirochetes (19).

Adhesion and invasion of vector cells by *B. burgdorferi* are important for both horizontal and vertical transmission and for transmission to mammalian hosts (9). Previous studies have indicated that this spirochete uses similar mechanisms to invade both mammalian and tick cells (9,22,23). The tick cell line derived from developing adult *R. appendiculatus* seems to use the same phagocytic mechanism that hemocytes use to phagocytose spirochetes, known as coiling phagocytosis (9,24,25).

The different levels of association between spirochetes and tick cell lines observed in the present study reinforce the findings that some embryo-derived tick cell lines do not present phagocytic features and consequent immune response against *Borrelia* (8). Characterization of the cell types in these cell lines is fundamental to the isolation and cultivation of field borreliae isolates (8,26). The cell lines used in the present study are heterogeneous, derived from multiple tick embryonic, larval or nymphal tissues (Table 1) and the cell types represented in each culture are largely unknown. The differences observed in invasion of different cell lines of the same tick species, namely *R. appendiculatus* RA243 and RAE/CTVM1 and *I. scapularis* IDE8 and ISE6, could be interpreted more as reflecting differences in the origin of the cells within cultures than different tick species (9,24).

In addition, the culture medium might influence phagocytic capacity. BSK-H medium is ideal for spirochetes (6,27) but not for tick cells, and higher concentrations of this medium might affect either the phagocytic capacity of tick cells or the ability of the spirochetes to penetrate the cells or both, resulting in a decrease in the percentage of fluorescent cells.

The cell line IRE/CTVM19 showed the lowest level of spirochete-cell association by flow cytometry for both medium combinations. This could be due to the factors discussed above, or to a higher level of resistance to attachment/penetration resulting from the natural interaction between *Ixodes* ticks and *B. burgdorferi* developed over many millenia (28). A similar inverse correlation between vector capacity and *in vitro* infectability has been reported for some intracellular tick-borne bacteria such as *Anaplasma marginale* and *Ehrlichia ruminantium*, both of which more readily infected cell lines derived from non-vector than vector tick species (13,29).

Confocal microscopy revealed that a small proportion of the bacteria were attached to the cell surface, while most of the fluorescence appeared inside the cells as variably sized points. Similar results were reported by Tuominen-Gustafsson and collaborators (19) for borreliae stained in human neutrophils.

Cytochalasin depolymerizes actin, causing distinct morphological changes, including the loss of pseudopodia. In other studies, cytochalasin effectively inhibited the phagocytosis of *Borrelia* spirochetes by cell lines derived from embryonic *I. scapularis* (IDE12) and *Dermacentor andersoni* (DAE15) (8). Tuominen-Gustafsson and collaborators (19) also obtained the same results by treating human neutrophils with cytochalasin. Our study confirmed these observations, showing a 75% reduction in intracellular spirochetes following cytochalasin treatment of the RA243 cell line.

One major concern in phagocytosis assays is to distinguish truly internalized bacteria from those that are only attached to the cell. In this study, microscopy data showed that small numbers of fluorescent bacteria were attached to the cell surface, and attachment could not be distinguished from phagocytosis by flow cytometry. Thus, the flow cytometry results should be interpreted as the association of bacteria with cells, including both attachment and uptake (19). Our data using trypan blue shows no difference in the mean fluorescence percentage, confirming that most spirochetes were inside the tick cells. On the other hand, in human blood phagocytes, a clear distinction between adherent extracellular spirochetes and ingested intracellular spirochetes is described (30).

In conclusion, staining *B. burgdorferi* with PKH is a valuable tool for analyzing the interactions between spirochetes and tick cells. Spirochetes labeled with PKH67 or PKH26 can be used for the quantitative analysis of their association with tick cells by flow cytometry, fluorescence and confocal microscopy.
